# Case report: One case of precise resection of gastric glomus tumor by gastroscopy combined with laparoscopy

**DOI:** 10.3389/fonc.2024.1501442

**Published:** 2025-01-07

**Authors:** Xuan Yang, Yunping Guo, Xiaochen Yan, Bin Xu, Zhenqin Cui, Zhuang Guo

**Affiliations:** ^1^ Department of Gastroenterology, Shengli Oilfield Central Hospital, Dongying, China; ^2^ Shengli Oilfield Central Hospital, Affiliated Binzhou Medical University, Dongying, Shandong, China; ^3^ Department of pathology, Shengli Oilfield Central Hospital, Dongying, China

**Keywords:** gastric glomus tumor, submucosal tumor, gastroscopy, laparoscopy, male

## Abstract

**Introduction:**

Gastric glomus tumor is a rare submucosal mesenchymal tumor with no distinct features on endoscopy. In clinical practice, it is often treated with laparoscopic partial gastrectomy. Here, we report a case of gastric glomus tumor successfully resected using a combination of gastroscopy and laparoscopy.

**Case description:**

The patient was an elderly male who underwent gastroscopy, which revealed a submucosal mass in the gastric antrum. The lesion was suspected to be a stromal tumor. Further evaluation with computed tomography (CT) imaging indicated a space-occupying lesion in the gastric antrum, with the possibility of benign pathology. Endoscopic ultrasonography revealed that the tumor originated from the muscular layer and was approximately 28.8 mm ×22.5 mm. Blood flow was detected behind the lesion, suggesting the possibility of a gastric glomus tumor. The tumor was removed using a combination of gastroscopy and laparoscopy. Postoperative pathology suggested that it was a benign gastric glomus tumor. The patient recovered uneventfully and was discharged 7 days later.

**Conclusion:**

Gastric glomus tumor is a rare submucosal tumor that should be included in the differential diagnosis of gastric submucosal tumors. A combined approach using gastroscopy and laparoscopy offers a minimally invasive and effective treatment option.

## Introduction

A glomus tumor is a rare mesenchymal neoplasm originating from the neuromuscular arterial canal or glomus body. While common in the extremities, it is rarely found in internal organs. However, tumors are reported in the mediastinum, trachea, kidneys, uterus, vagina, and stomach (1–3). Gastric glomus tumors (GGT) typically occur in the antrum or pylorus, involving the muscularis propria or submucosa. GGT accounts for only 1% of stromal tumors in the stomach (4). GGT can manifest as indigestion, upper abdominal pain, nausea, vomiting, hematemesis, or melena in clinical practice. However, they lack specific clinical manifestations, and some cases are asymptomatic (5). Owing to the lack of specific clinical and endoscopic features, GGT is often misdiagnosed as more common gastrointestinal stromal tumors (6). Here, we report a case of a GGT resected using a combination of endoscopy and laparoscopy.

## Case description

**Figure 1:**
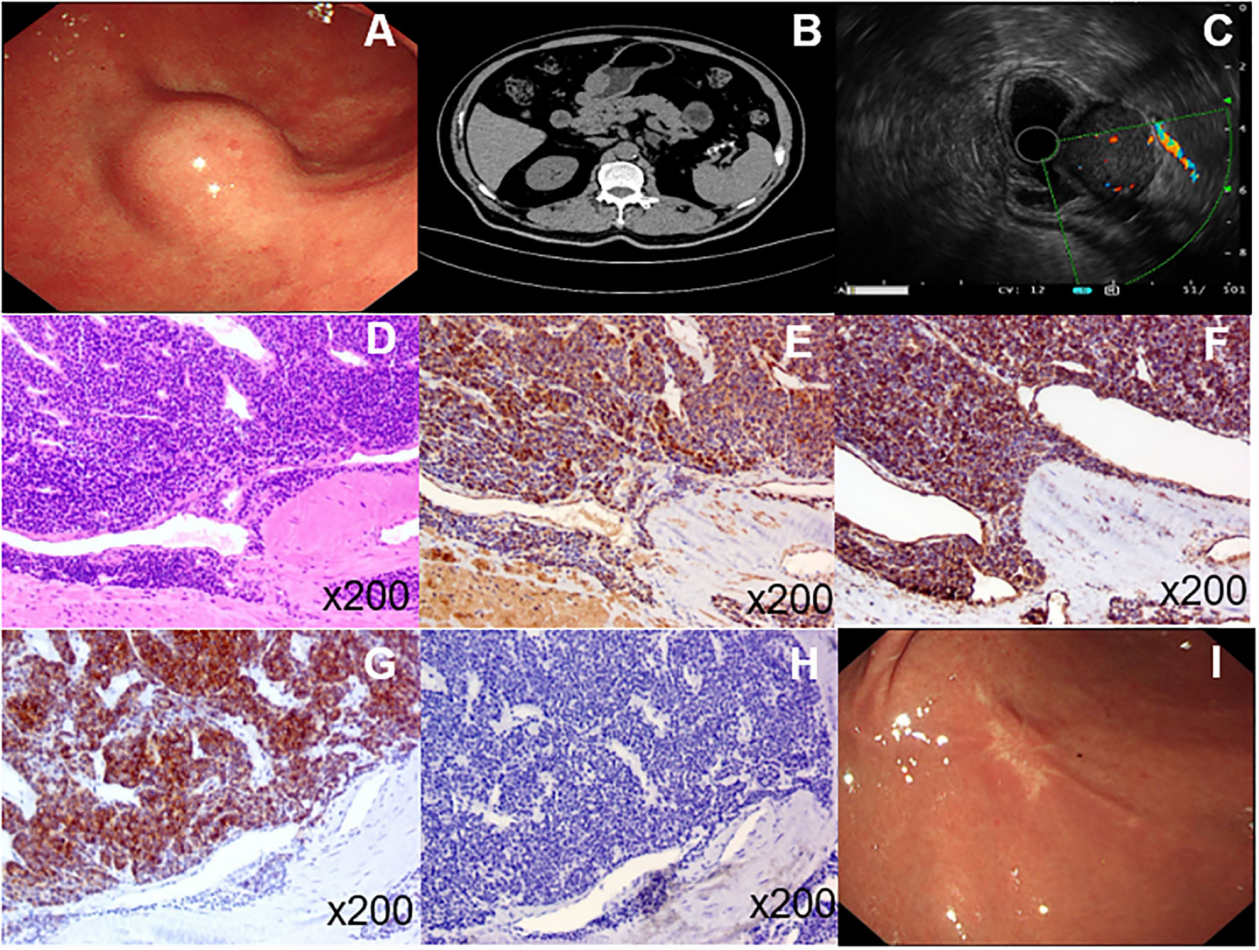
**(A)**, Endoscopy of the upper digestive tract revealed a submucosal tumor in the gastric antrum. **(B)**, CT imaging identified a space-occupying lesion in the gastric antrum. **(C)**, Endoscopic ultrasonography showed that the tumor originated from the muscular layer with minimal blood flow inside and detectable blood vessels behind it. Under the microscope, the tumor appeared to be composed of aggregated round and spindle-shaped cells surrounded by capillaries **(D)**. Immunohistochemistry revealed positive expression of smooth muscle actin and vimentin **(E, F)**; however, no expression of syn and C-kit **(G, H)**. The patient was re-examined by gastroscopy 1 year after the operation, and the wound healed well **(I)**.

The patient was an elderly male without any underlying diseases. A routine health checkup revealed a submucosal bulge in the gastric antrum during gastroscopy (**Figure 1A**). We conducted tests on blood routine, liver and kidney function, electrolytes, and gastrointestinal tumor markers, all of which showed no significant abnormalities. Further improvement of the CT scan indicated an occupied lesion in the gastric antrum, which was considered benign (**Figure 1B**). Endoscopic ultrasonography (EUS) revealed that the tumor originated from the muscle layer and measured approximately 28.8 × 22.5 mm. A small amount of blood flow was observed within the tumor, and blood vessels behind it were also visualized (**Figure 1C**). The tumor exhibited mixed echogenicity, with a clear boundary and grew inside and outside the gastric cavity. We considered the tumor to be more of a gastric stromal tumor; however, glomus tumors and schwannomas could not be excluded. To ensure accurate removal, laparoscopy combined with gastroscopy was performed. First, an endoscopic gastric tumor resection was conducted under general anesthesia with tracheal intubation. During the procedure, a 30 mm bulge was observed on the greater curvature of the gastric antrum. Using a Dual knife, we incised the mucosa down to the muscularis propria, exposed the tumor, gradually dissected it to reveal most of its structure, and then proceeded with laparoscopic resection. Under the laparoscope, a 5 mm perforation was observed approximately 30 mm from the anterior wall of the gastric antrum, surrounded by a small amount of bloody effusion. An ultrasonic scalpel was used to carefully dissect the edge of the tumor and the perforation site while ensuring the gastric wall was protected. After surgery, the size of the tumor was approximately 30 mm × 30 mm, and the capsule remained intact. Histopathological analysis revealed that the tumor was clearly confined to the submucosa. Microscopic examination revealed that it consisted of clusters of round and spindle-shaped cells surrounded by capillaries (**Figure 1D**). Immunohistochemistry revealed positive expression of smooth muscle actin and vimentin (**Figures 1E, F**) but negative expression of synaptophysin (Syn) and C-kit (**Figures 1G, H**). Based on these findings, the tumor was diagnosed as a GGT. After surgery, patients were restricted from eating, and a gastric tube was used for drainage and decompression. Nutritional and electrolyte support was administered intravenously to meet their basic physiological needs. After 3 days, the patient transitioned to a liquid diet and was discharged after 7 days. The patient was followed up at our hospital 1year after the operation. No abdominal pain, abdominal distension, or other discomfort were observed. Gastroscopy was performed again, and the analysis revealed that the surgical wound was perfectly healed (**Figure 1I**).

Glomus tumor is a rare mesenchymal tumor originating from the neuromuscular arterial canal or glomus. Gastrointestinal glomus tumors are rare within the stomach, particularly the gastric antrum, being the most common site of involvement. These tumors typically range in size from 8–110 mm and are often misdiagnosed as the more common gastrointestinal stromal tumors. It is often difficult to diagnose gastric glomus tumors using conventional imaging examinations, such as computed tomography and magnetic resonance imaging (7). EUS clearly identifies the origin of the lesion, reveals the stratification of the digestive tract wall, and provides information on echo intensity, echo uniformity, and the boundary of the lesion. Despite these advantages, the endoscopic ultrasound appearance of glomus tumor has similar manifestations with other submucosal tumors, which are often difficult to identify (**Table 1**) (2, 8). In color Doppler images, blood flow within the glomus tumor is sometimes visible. However, when the blood flow velocity is low, or the acoustic angle is suboptimal, blood flow is not well displayed or entirely absent. Endoscopic ultrasound-guided fine-needle aspiration (EUS-FNA) is a reliable method for obtaining pathological specimens from gastric submucosal tumors. However, for tumors exhibiting malignant characteristics (e.g., necrotic changes and rapid growth), EUS-FNA should be performed cautiously to minimize the risk of tumor cell metastasis through the needle tract (9, 10). In this case report, the tumor of the patient was large. A high-frequency-probe was used during EUS to repeatedly adjust the observation settings. The tumor appeared as a low-echo mass originating from the third or fourth submucosal layer, with a clear boundary; however, uneven internal echo. A dot-like strong echo was observed within the tumor, although no significant blood flow signals were detected. We recommended that the patient undergo EUS-FNA for further examination; however, the patient declined. The tumor was suspected to be a stromal tumor; however, the possibility of a glomus tumor could not be ruled out.

**Table 1:** Characteristic features of gastric submucosal mass lesions.

Tumor	Disease	Predominant gastric layer(s)	Specific findings on EUS
Mesenchymal tumor	(Gastrointestinal stromal tumor (GIST)	Muscularis propria layer	Hypoechoic; typically fourth layer, rarely second layer (muscularis mucosa)
	Leiomyoma	Muscularis propria layer	Hypoechoic; fourth layer, rarely second layer (muscularis mucosa)
	Schwannoma	Muscle	Low echo; fourth layer
	Lipoma	Submucosa	Homogeneous, hyperechoic, smooth, oval, third layer
	Vascular tumor (i.e.hoemangioma)	Submucosa	Varied echo patterns from high to low; third to fourth layers
	Lymphatic tumor (i.e.lymphangiomo)	Submucosa	Multiple anechoic lesions; third layer
	Inflammatory Fibroid tumor (i.e.Brunner’s gland hamartoma)	Mucosa and submucosa	Heterogenecus echo; second layer
Lymphoma	MALT lymphoma	Mucosa and submucosa	Low echo; second and third layers
	Malignant lymphoma	Mucosa and submucosa	Low echo; second and third layers

The National Comprehensive Cancer Network guidelines recommend EUS-guided fine-needle aspiration or core needle biopsy for undiagnosed gastric submucosal tumors < 2 mm diameter (11, 12). Surgical resection is recommended if high-risk features are present —such as tumor size (> 30 mm), irregular margins, regional lymph node involvement, and tumor rupture as identified by EUS—surgical resection is advised (13). Surgical approaches vary, ranging from traditional complex organ and gastric wedge resections to advanced techniques such as endoscopic full-thickness resections, endoscopic submucosal dissections, and combined endoscopic/laparoscopic procedures (14, 15). Currently, an increasing number of clinicians and patients prefer endoscopic surgery owing to its minimal trauma and faster recovery (16, 17). The exact nature of the gastric antrum tumor in this patient remains unclear. We consider the prospect of a stromal tumor to be high; however, the possibility of a glomus tumor cannot be excluded. Based on enhanced CT and EUS, the tumor was observed to grow inside and outside the gastric cavity, with a relatively large size and a rich blood supply. We identified three major problems in managing the tumor through simple gastroscopic procedures. First, the significant growth of the tumor outside the cavity obscures the visualization of external blood vessels, creating a poor field of view and making bleeding difficult to control if it occurs. Second, the large size of the tumor and adherence to the muscular layer made perforation inevitable during the dissection process. Moreover, the perforation area was extensive, making it difficult to seal effectively using endoscopic techniques. Third, if the tumor margin were positive, additional surgery would be necessary. Therefore, we opted for a combined gastroscopy and laparoscopy approach to ensure complete resect tumor removal. First, the intracavitary portion of the tumor was dissected endoscopically, followed by laparoscopic removal of the extracavitary growth. This method allowed full visualization of the intracavitary and extracavitary components, minimized accidental injury and unnecessary resection, and enabled precise excision. Pathological tests confirmed the tumor as a benign gastric glomus tumor with negative surgical margins. After surgery, the patient underwent acid suppression, rehydration, fasting, nutritional support, and other treatments. On the first postoperative day, the patient was mobilized out of bed. After 3 days, oral intake was gradually resumed. The patient showed steady recovery and was discharged after 1 week.

## Conclusion

GGT are rare submucosal lesions that should be considered in the differential diagnosis of gastric submucosal tumors. While EUS offers valuable insights for the diagnosis of submucosal tumors, immunohistochemical analysis remains essential. Gastroscopy combined with laparoscopic resection is an accurate, effective, and safe treatment option for glomus tumors.

## Data Availability

The original contributions presented in the study are included in the article/supplementary material. Further inquiries can be directed to the corresponding author/s.
